# Editorial: Molecular genetics of cutaneous melanoma: current status and future direction-volume II

**DOI:** 10.3389/fmolb.2023.1273849

**Published:** 2023-08-31

**Authors:** Zaida Garcia-Casado, Cristina Pellegrini, William C. Cho

**Affiliations:** ^1^ Laboratory of Molecular Biology, Instituto Valenciano de Oncologia, Valencia, Spain; ^2^ Dermatology, Department of Biotechnological and Applied Clinical Sciences, University of L’Aquila, L’Aquila, Italy; ^3^ Department of Clinical Oncology, Queen Elizabeth Hospital, Kowloon, Hong Kong SAR, China

**Keywords:** cutaneus melanoma, biomarkers, immunotherapy, prognosis, emerging targets

The incidence of malignant melanoma is rising steadily, and advanced melanoma remains one of the most lethal forms of cancer (Sung et al., 2021). The development of immune checkpoint inhibitors and targeted therapies has prolonged the survival of melanoma patients (Rutkowski and Czarnecka, 2023; Qin and Zheng, 2023; Rizzetto et al., 2023). However, drug resistance develops in the majority of patients and new strategies with a significant impact on the treatment outcome are in demand. This points to the need for robust predictive biomarkers and new rational targets for more effective combination therapies.

A major problem in cancer treatment, particularly with the use of conventional chemotherapy, is the emergence of small subpopulations of cells called persister cells (Ramirez et al., 2016), which are associated with the occurrence of relapse. In the case of melanoma, these therapies are not often used due to high recurrence rates. Karki et al. defined the metabolic mechanisms of melanoma persister cells derived from gemcitabine-treated melanoma cell cultures. They concluded that chemotherapeutic agents promote the formation of persister cells. Furthermore, metabolic profiling of melanoma persister cells revealed that the metabolic alteration was associated with an upregulation of the utilisation of Krebs cycle metabolites and this appeared to be independent of drug concentration and treatment time. Hence, they point the way to targeting persister cell metabolism as a therapeutic strategy.

To address the issue of low efficacy of immunotherapy in melanoma, Lin et al. hypothesized that estrogen may affect the tumor microenvironment (TME), thereby influencing its response to this therapy. Using RNA sequencing data from 4 immunotherapy-treated melanoma datasets and TCGA data of melanoma, they constructed an 11-gene estrogen response-related signature to predict response to immunotherapy in melanoma. They also investigated the feasibility of combining endocrine therapy with immunotherapy. In the same direction, Xu and Guo presented FCGR1A, encoding the CD64 transmembrane glycoprotein, as a novel potential biomarker in four types of cancer (cervical and endocervical cancer, cholangiocarcinoma, kidney renal clear cell carcinoma and skin cutaneous melanoma). Xu and Guo showed that FCGR1A expression may be a potential biomarker in cancer with a relevant role in tumor immunology and correlated with immune cells and several immune cell marker genes. FCGR1A expression was associated with prognosis in these four cancer types, including melanoma, where high expression correlated with better overall survival (HR: 0.53, *p* = 3.2e-06). These results suggested that FCGR1A may play an important role in immune cell infiltration and may serve as a prognostic biomarker, particularly in melanoma.

Recognizing the need to develop new methods to identify markers of immunotherapy response and characterize the tumor immune microenvironment in cancer patients, Yaseen et al. validated a 6-plex immunofluorescence panel of immune cell markers CD8, CD68, CD16, the immune checkpoint PD-L1 and the melanoma tumor marker SOX10 in 67 metastatic melanoma FFPE specimens from 67 patients. The authors found a high level of accuracy, precision and reproducibility of this multiplex immunofluorescence staining in comparison with immunohistochemistry and single-plex immunofluorescence. The technique preserves the integrity and histological location of immune, resident and tumor cells in tissue sections and allows the co-localisation of multiple markers. This will be particularly useful for the identification of predictive biomarkers in melanoma patients on immunotherapy.

Alongside the studies focused on finding new prognostic biomarkers, an increasing interest is directed toward the epigenetics changes associated with melanoma progression, response to treatments and resistance. As reviewed by Wozniak and Czyz, epigenetic alterations have been identified as novel targets for the treatment of patients with melanoma. They focused on the role of EZH2 in melanoma progression and how lncRNAs modulate its expression and activity. EZH2 is frequently amplified, overexpressed and activated in melanoma. This correlates with DNA methylation and epigenetic silencing of melanoma tumor suppressors. In addition, EZH2 is involved in the regulation of the TME and the antitumor immune response, leading to a reduction in the efficacy of immunotherapy. On the other hand, recent data confirm the contribution of EZH2 to resistance to targeted therapy against BRAF V600 and MEK1/2 in melanoma. As a result, EZH2 is becoming an emerging target for the treatment of various solid cancers, including melanoma.

The collection of articles on this Research Topic ranges from research articles (3) to methods (1) and review (1) articles on the identification of novel biomarkers and potential targets in melanoma. These studies are an important contribution to the field of “*Molecular Genetics of Cutaneous Melanoma*”, as denoted by the title of the Research Topic.
